# Supplementary material for: Beyond mono fertilization: Mixed fertilization enhances productivity and quality of chili (Capsicum frutescens)
Source: PLoS One. 2026 Mar 31;21(3):e0331538. doi: 10.1371/journal.pone.0331538 (PMC13037990; doi:10.1371/journal.pone.0331538)
Supplement: S1 File — (DOCX) [file pone.0331538.s001.docx]

**R scripts for the figures**

**Plant height (a)**

ggplot(tgc, aes(x=T, y=PH, fill=DI))+ geom_bar(position=position_dodge(0.8),width = 0.5, stat="identity")+theme_bw()+ theme(panel.grid.major = element_blank(), panel.grid.minor = element_blank())+geom_errorbar(aes(ymin=PH-se, ymax= PH+se), width=.20, position=position_dodge(0.8))+xlab("Treatments")+ylab("Plant height (cm)") +theme(axis.text=element_text(size=10,face='bold'))+theme(axis.title.y = element_text(vjust = +2))+theme(axis.title.x = element_text(vjust = -1))+ theme(legend.position=c(0.11,0.88))+ theme(legend.key.width=unit(0.25,"cm"),legend.key.height=unit(0.15,"cm"))+ theme(legend.text = element_text(size= 7.5))+theme(axis.title = element_text(face="bold", size=10))+ theme(legend.title = element_blank())+theme(axis.title.x = element_blank())+ geom_vline(xintercept=1.5, linetype="dashed", color = "black", size=0.8)+ geom_vline(xintercept=3.5, linetype="dashed", color = "black", size=0.8)+ylim(0,100)+ annotate("text",x= 0.7, y = 36, label = "b", size=3, color='black')+ annotate("text",x= 0.9, y = 51, label = "ab", size=3, color='black')+ annotate("text",x= 1.1, y = 64, label = "a", size=3, color='black')+ annotate("text",x= 1.3, y = 70, label = "a", size=3, color='black')+ annotate("text",x= 1.7, y = 32, label = "a", size=3, color='black')+ annotate("text",x= 1.9, y = 40, label = "a", size=3, color='black')+ annotate("text",x= 2.1, y = 47, label = "a", size=3, color='black')+ annotate("text",x= 2.3, y = 54, label = "a", size=3, color='black')+ annotate("text",x= 2.7, y = 38, label = "a", size=3, color='black')+ annotate("text",x= 2.9, y = 52, label = "a", size=3, color='black')+ annotate("text",x= 3.1, y = 57, label = "a", size=3, color='black')+ annotate("text",x= 3.3, y = 62, label = "a", size=3, color='black')+ annotate("text",x=3.7, y = 40, label = "b", size=3, color='black')+ annotate("text",x= 3.9, y = 58, label = "ab", size=3, color='black')+ annotate("text",x= 4.1, y = 67, label = "a", size=3, color='black')+ annotate("text",x= 4.3, y = 73, label = "a", size=3, color='black')+ annotate("text",x=4.7, y = 44, label = "b", size=3, color='black')+ annotate("text",x=4.9, y = 69, label = "ab", size=3, color='black')+ annotate("text",x=5.1, y = 80, label = "a", size=3, color='black')+ annotate("text",x=5.3, y = 87, label = "a", size=3, color='black')+ annotate("text",x=1, y =76, label = "B", size=4.5, color='navy blue')+ annotate("text",x=2.5, y = 70, label = "B", size=4.5, color='navy blue')+ annotate("text",x=4.5, y = 95, label = "A", size=4.5, color='navy blue') +scale_fill_brewer(palette = "BrBG")

**Number of leaves (b)**

ggplot(tgc, aes(x=T, y=NL, fill=DI))+ geom_bar(position=position_dodge(0.8),width = 0.5, stat="identity")+theme_bw()+ theme(panel.grid.major = element_blank(), panel.grid.minor = element_blank())+geom_errorbar(aes(ymin=NL-se, ymax= NL+se), width=.20, position=position_dodge(0.8))+xlab("Treatments")+ labs(y=expression(bold('Number of leaves'~(plant^-1)))) +theme(axis.text=element_text(size=10,face='bold'))+theme(axis.title.y = element_text(vjust = +2))+theme(axis.title.x = element_text(vjust = -1))+ theme(legend.position=c(0.1,0.88))+ theme(legend.key.width=unit(0.45,"cm"),legend.key.height=unit(0.25,"cm"))+ theme(legend.text = element_text(size= 10))+theme(legend.title = element_blank())+theme(axis.title.x = element_blank())+ geom_vline(xintercept=1.5, linetype="dashed", color = "black", size=0.8)+ geom_vline(xintercept=3.5, linetype="dashed", color = "black", size=0.8)+ theme(axis.title = element_text(face="bold", size=10))+ annotate("text",x= 0.7, y = 290, label = "c", size=3, color='black')+ annotate("text",x= 0.9, y = 580, label = "b", size=3, color='black')+ annotate("text",x= 1.1, y = 820, label = "a", size=3, color='black')+ annotate("text",x= 1.3, y = 950, label = "a", size=3, color='black')+ annotate("text",x= 1.7, y = 190, label = "d", size=3, color='black')+ annotate("text",x= 1.9, y = 315, label = "c", size=3, color='black')+ annotate("text",x= 2.1, y = 445, label = "b", size=3, color='black')+ annotate("text",x= 2.3, y = 590, label = "a", size=3, color='black')+ annotate("text",x= 2.7, y = 300, label = "c", size=3, color='black')+ annotate("text",x= 2.9, y = 605, label = "b", size=3, color='black')+ annotate("text",x= 3.1, y = 695, label = "ab", size=3, color='black')+ annotate("text",x= 3.3, y = 750, label = "a", size=3, color='black')+ annotate("text",x=3.7, y = 400, label = "c", size=3, color='black')+ annotate("text",x= 3.9, y = 750, label = "b", size=3, color='black')+ annotate("text",x= 4.1, y = 940, label = "ab", size=3, color='black')+ annotate("text",x= 4.3, y = 1060, label = "a", size=3, color='black')+ annotate("text",x=4.7, y = 395, label = "d", size=3, color='black')+ annotate("text",x=4.9, y = 755, label = "c", size=3, color='black')+ annotate("text",x=5.1, y = 955, label = "b", size=3, color='black')+ annotate("text",x=5.3, y = 1095, label = "a", size=3, color='black')+ylim(0,1250)+ annotate("text",x=1, y = 1020, label = "B", size=4.5, color='navy blue')+ annotate("text",x=2.5, y = 800, label = "B", size=4.5, color='navy blue')+ annotate("text",x=4.6, y = 1160, label = "A", size=4.5, color='navy blue') +scale_fill_brewer(palette = "BrBG")+theme(legend.position = "none")

**Number of flowering (c)**

ggplot(tgc, aes(x=T, y=NF, fill=DI))+ geom_bar(position=position_dodge(0.8),width = 0.5, stat="identity")+theme_bw()+ theme(panel.grid.major = element_blank(), panel.grid.minor = element_blank())+geom_errorbar(aes(ymin=NF-se, ymax= NF+se), width=.20, position=position_dodge(0.8))+xlab("Treatments")+labs(y=expression(bold('Number of flowering'~(plant^-1)))) +theme(axis.text=element_text(size=10,face='bold'))+theme(axis.title.y = element_text(vjust = +2))+theme(axis.title.x = element_text(vjust = -1))+ theme(legend.position=c(0.1,0.9))+ theme(legend.key.width=unit(0.25,"cm"),legend.key.height=unit(0.25,"cm"))+ theme(legend.text = element_text(size= 9))+theme(legend.title = element_blank())+theme(axis.title.x = element_blank())+ geom_vline(xintercept=1.5, linetype="dashed", color = "black", size=0.8)+ geom_vline(xintercept=3.5, linetype="dashed", color = "black", size=0.8)+theme(axis.title = element_text(face="bold", size=10))+ylim(0,65)+annotate("text",x=0.8, y = 23, label = "a", size=3, color='black')+annotate("text",x=1.2, y = 39, label = "a", size=3, color='black')+annotate("text",x=1.8, y = 16, label = "b", size=3, color='black')+annotate("text",x=2.2, y = 28, label = "a", size=3, color='black')+annotate("text",x=2.8, y = 19, label = "b", size=3, color='black')+annotate("text",x=3.2, y = 31, label = "a", size=3, color='black')+annotate("text",x=3.8, y = 29, label = "b", size=3, color='black')+annotate("text",x=4.2, y = 45, label = "a", size=3, color='black')+annotate("text",x=4.8, y = 32, label = "a", size=3, color='black')+annotate("text",x=5.2, y = 59, label = "a", size=3, color='black')+annotate("text",x=0.9, y = 45, label = "B", size=4.5, color='navy blue')+annotate("text",x=2.5, y = 40, label = "B", size=4.5, color='navy blue') +annotate("text",x=4.5, y = 63, label = "A", size=4.5, color='navy blue')+theme(legend.position = "none")+scale_fill_manual(values=c("#dfc27d", "#80cdc1"))

**Number of fruits (d)**

ggplot(tgc, aes(x=T, y=NFr, fill=DI))+ geom_bar(position=position_dodge(0.8),width = 0.5, stat="identity")+theme_bw()+ theme(panel.grid.major = element_blank(), panel.grid.minor = element_blank())+geom_errorbar(aes(ymin=NFr-se, ymax= NFr+se), width=.20, position=position_dodge(0.8))+xlab("Treatments")+labs(y=expression(bold('Number of fruit'~(plant^-1)))) +theme(axis.text=element_text(size=10,face='bold'))+theme(axis.title.y = element_text(vjust = +2))+theme(axis.title.x = element_text(vjust = -1))+ theme(legend.position=c(0.105,0.88))+ theme(legend.key.width=unit(0.35,"cm"),legend.key.height=unit(0.10,"cm"))+ theme(legend.text = element_text(size= 8.5))+theme(legend.position = "none")+theme(legend.title = element_blank())+theme(axis.title.x = element_blank())+ geom_vline(xintercept=1.5, linetype="dashed", color = "black", size=0.8)+ geom_vline(xintercept=3.5, linetype="dashed", color = "black", size=0.8)+theme(axis.title = element_text(face="bold", size=10))+ ylim(0,120)+ annotate("text",x=0.73, y = 7, label = "b", size=3, color='black')+ annotate("text",x=1, y = 27, label = "b", size=3, color='black')+ annotate("text",x=1.27, y = 62, label = "a", size=3, color='black')+ annotate("text",x=1.73, y = 7, label = "b", size=3, color='black')+ annotate("text",x=2, y = 23, label = "b", size=3, color='black')+ annotate("text",x=2.27, y = 51, label = "a", size=3, color='black')+ annotate("text",x=2.73, y = 14, label = "c", size=3, color='black')+ annotate("text",x=3, y = 27, label = "b", size=3, color='black')+ annotate("text",x=3.27, y = 55, label = "a", size=3, color='black')+ annotate("text",x=3.73, y = 14, label = "c", size=3, color='black')+ annotate("text",x=4, y = 37, label = "b", size=3, color='black')+ annotate("text",x=4.27, y = 73, label = "a", size=3, color='black')+ annotate("text",x=4.73, y = 28, label = "c", size=3, color='black')+ annotate("text",x=5, y = 54, label = "b", size=3, color='black')+ annotate("text",x=5.27, y = 106, label = "a", size=3, color='black')+ annotate("text",x=0.9, y = 75, label = "B", size=4.5, color='Navy Blue')+ annotate("text",x=2.5, y = 75, label = "B", size=4.5, color='Navy Blue')+ annotate("text",x=4.5, y = 110, label = "A", size=4.5, color='Navy Blue')+scale_fill_manual(values=c("#dfc27d", "#80cdc1","#018571"))


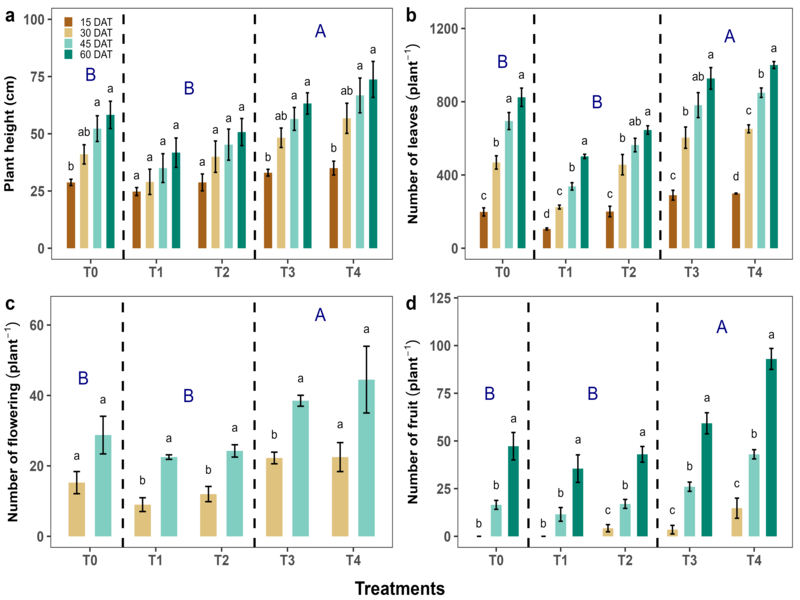


**Fig 3. Effect of different fertilization treatments on plant height, number of leaves, flowering and fruit production of *Capsicum frutescens* at various DAT.**

**Above ground net primary productivity (ANPP) (a)**

qplot(data = AT, x = T, y = ANPP, fill = T, geom = "boxplot")+theme_bw()+labs(x=expression(bold('Treatments')))+ stat_boxplot(geom = "errorbar", width = 0.2, position=position_dodge(0.8))+labs(y=expression(bold('ANPP'~(g~plant^-1))))+ theme(panel.grid.major = element_blank(), panel.grid.minor = element_blank())+theme(axis.text=element_text(size=10,face='bold'))+theme(legend.position=c(0.1,0.9))+ theme(legend.key.width=unit(0.50,"cm"),legend.key.height=unit(0.50,"cm"))+ theme(legend.text = element_text(size= 8.5))+theme(legend.title = element_blank())+theme(legend.position = "top")+ geom_vline(xintercept=1.5, linetype="dashed", color = "black", size=0.8)+ geom_vline(xintercept=3.5, linetype="dashed", color = "black", size=0.8)+theme(axis.text.x=element_blank())+theme(axis.title.y = element_text(vjust = +2))+theme(axis.title.x = element_blank())+theme(axis.ticks.x=element_blank())+ stat_summary(fun.y = mean, color = "white", position = position_dodge(0.75), geom = "point", shape = 18, size = 2) +scale_fill_manual(values=c("#d8b709", "darkolivegreen3","#a2a475","#0c775e","#972d14"))+ylim(0,50)+theme(axis.title = element_text(face="bold", size=11))+ annotate("text",x=c(1,2.5,4.5), y =c(48,48,48), label = c("B","C","A"),size=c(4.5,4.5,4.5), color='Navy Blue')+annotate("segment", x = 2, xend = 3, y = 25, yend = 25, size = 0.5, color = "black", linetype = "solid")+annotate("segment", x = 2, xend = 2, y = 25, yend = 23, size = 0.5)+annotate("segment", x = 3, xend = 3, y = 25, yend = 23, size = 0.5)+annotate("segment", x = 4, xend = 5, y = 40, yend = 40, size = 0.5, color = "black", linetype = "solid")+annotate("segment", x = 4, xend = 4, y = 40, yend = 38, size = 0.5)+annotate("segment", x = 5, xend = 5, y = 40, yend = 38, size = 0.5)+annotate("text", x = c(2.5,4.5), y = c(28,43),label = c("mean: 10.08", "mean: 21.82") , color="black", size=3.5 , angle=0 ,fontface="bold")+annotate("text", x = c(1,2,3,4,5), y = c(28,14,19,29,34),label = c("ab", "c", "bc", "a", "a") , color="black", size=3.5 , angle=0 ,fontface="bold")

**Below ground net primary productivity (BNPP) (b)**

qplot(data = BT, x = T, y = BNPP, fill = T, geom = "boxplot")+theme_bw()+labs(x=expression(bold('Treatments')))+stat_boxplot(geom = "errorbar", width = 0.2, position=position_dodge(0.8))+labs(y=expression(bold('BNPP'~(g~plant^-1))))+ theme(panel.grid.major = element_blank(), panel.grid.minor = element_blank())+theme(axis.text=element_text(size=10,face='bold'))+theme(legend.position=c(0.1,0.9))+ theme(legend.key.width=unit(0.50,"cm"),legend.key.height=unit(0.50,"cm"))+ theme(legend.text = element_text(size= 8.5))+theme(legend.title = element_blank())+theme(legend.position = "top")+ geom_vline(xintercept=1.5, linetype="dashed", color = "black", size=0.8)+ geom_vline(xintercept=3.5, linetype="dashed", color = "black", size=0.8)+theme(axis.text.x=element_blank())+theme(axis.title.y = element_text(vjust = +2))+theme(axis.title.x = element_blank())+theme(axis.ticks.x=element_blank())+ stat_summary(fun.y = mean, color = "white", position = position_dodge(0.75), geom = "point", shape = 18, size = 2) +scale_fill_manual(values=c("#d8b709", "darkolivegreen3","#a2a475","#0c775e","#972d14"))+ylim(0,8)+theme(axis.title = element_text(face="bold", size=11))+ annotate("text",x=c(1,2.5,4.5), y =c(8,8,8), label = c("A","B","A"),size=c(4.5,4.5,4.5), color='Navy Blue')+annotate("segment", x = 2, xend = 3, y = 4.8, yend = 4.8, size = 0.5, color = "black", linetype = "solid")+annotate("segment", x = 2, xend = 2, y = 4.8, yend = 4.5, size = 0.5)+annotate("segment", x = 3, xend = 3, y = 4.8, yend = 4.5, size = 0.5)+ annotate("segment", x = 4, xend = 5, y = 6.8, yend = 6.8, size = 0.5, color = "black", linetype = "solid")+annotate("segment", x = 4, xend = 4, y = 6.8, yend = 6.5, size = 0.5)+annotate("segment", x = 5, xend = 5, y = 6.8, yend = 6.5, size = 0.5)+annotate("text", x = c(2.5,4.5), y = c(5.2,7.2),label = c("mean: 1.95", "mean: 3.93") , color="black", size=3.5 , angle=0 ,fontface="bold")+annotate("text", x = c(1,2,3,4,5), y = c(4.3,2.5,3.8,5.1,5.9),label = c("ab", "c", "bc", "ab", "a") , color="black", size=3.5 , angle=0 ,fontface="bold")

**Net primary productivity (NPP) (c)**

qplot(data = NT, x = T, y = NPP, fill = T, geom = "boxplot")+theme_bw()+labs(x=expression(bold('Treatments')))+ ylab(expression(bold(atop("NPP (ANPP + BNPP)", paste("(g ", plant^{-1}, ")")))))+ theme(panel.grid.major = element_blank(), panel.grid.minor = element_blank())+theme(axis.text=element_text(size=10,face='bold'))+theme(legend.position=c(0.1,0.9))+ theme(legend.key.width=unit(0.50,"cm"),legend.key.height=unit(0.50,"cm"))+ theme(legend.text = element_text(size= 8.5))+theme(legend.title = element_blank())+theme(legend.position = "top")+ geom_vline(xintercept=1.5, linetype="dashed", color = "black", size=0.8)+ geom_vline(xintercept=2.5, linetype="dashed", color = "black", size=0.8)+theme(axis.title.y = element_text(vjust = +1))+theme(axis.title.x =element_blank())+ stat_summary(fun.y = mean, color = "white", position = position_dodge(0.75), geom = "point", shape = 18, size = 2)+ scale_fill_manual(values=c("#d8b709","#0c775e","#972d14"))+ylim(0,50)+theme(axis.title = element_text(face="bold", size=11))+theme(legend.position="top")+annotate("text",x=c(1,2,3), y =c(45,45,45), label = c("AB","B","A"),size=c(4.5,4.5,4.5), color='Navy Blue')+theme(axis.text.x = element_blank())+theme(axis.ticks.x=element_blank())

**
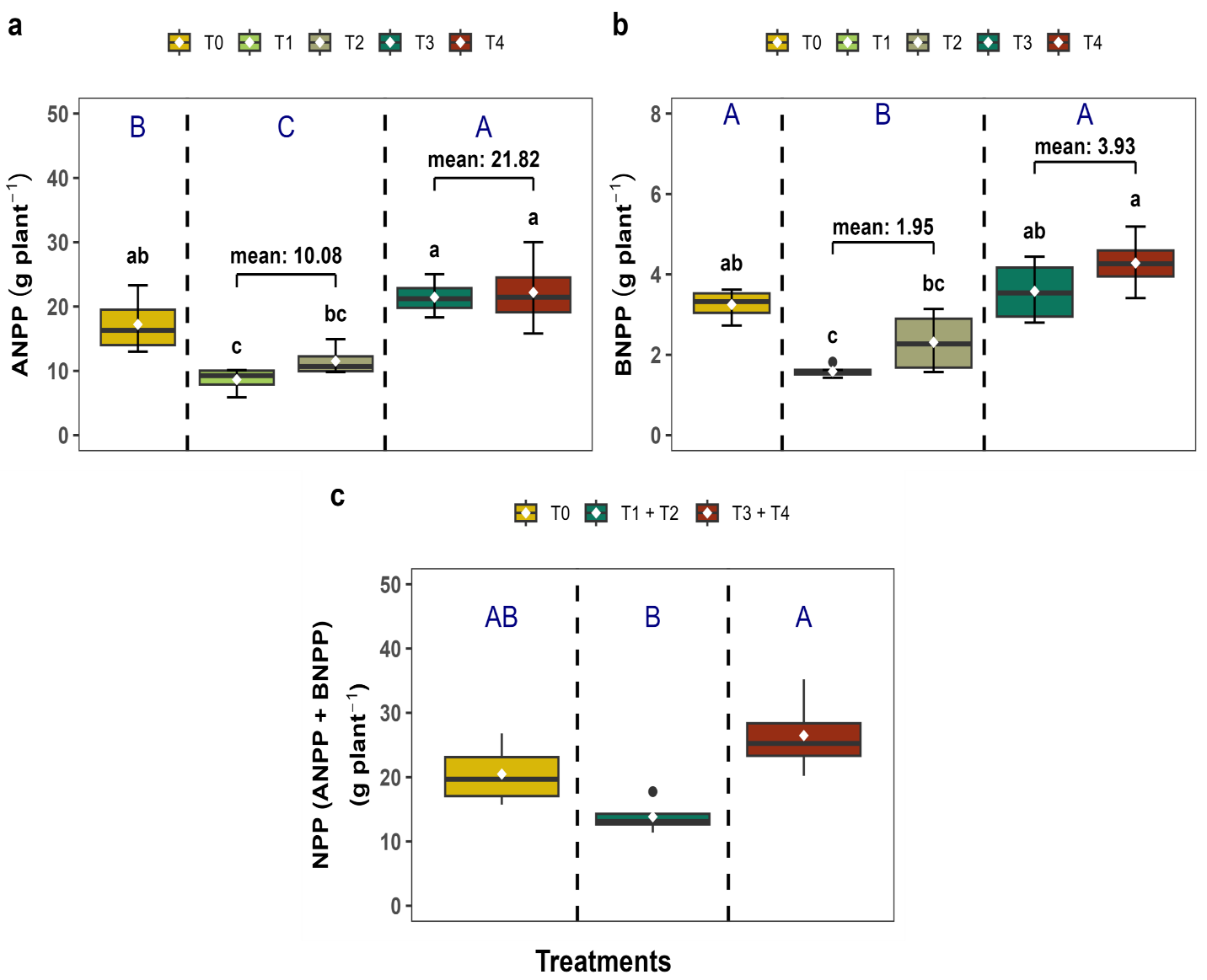
**

**Fig 4. (a) Aboveground Net Primary Productivity (ANPP), (b) Belowground Net Primary Productivity (BNPP) and (c) Net Primary Productivity (ANPP + BNPP) of *Capsicum frutescens* under different fertilization treatments.**

**Yield**

ggplot(tgc, aes(x=T, y=Y, fill=T))+ geom_bar(position=position_dodge(0.8),width = 0.5, stat="identity")+theme_bw()+ theme(panel.grid.major = element_blank(), panel.grid.minor = element_blank())+geom_errorbar(aes(ymin=Y-se, ymax= Y+se), width=.20, position=position_dodge(0.8))+xlab("Treatments")+labs(x=expression(bold('Treatments')))+labs(y=expression(bold('Yield'~(t~ha^-1)))) +theme(axis.text=element_text(size=10,face='bold'))+theme(axis.title.y = element_text(vjust = +2))+theme(axis.title.x = element_text(vjust = -1))+theme(legend.position=c(0.1,0.9))+ theme(legend.key.width=unit(0.50,"cm"),legend.key.height=unit(0.50,"cm"))+ theme(legend.text = element_text(size= 8.5))+theme(legend.title = element_blank())+theme(legend.position = "top")+ geom_vline(xintercept=1.5, linetype="dashed", color = "black", size=0.8)+ geom_vline(xintercept=3.5, linetype="dashed", color = "black", size=0.8)+theme(axis.text.x=element_blank())+theme(axis.title.y = element_text(vjust = +2))+theme(axis.title.x = element_text(vjust = 0))+theme(axis.ticks.x=element_blank())+ scale_fill_brewer(palette = "BrBG") +ylim(0,25)+theme(axis.title = element_text(face="bold", size=13))+ annotate("text",x=c(1,2.5,4.5), y =c(25,25,25), label = c("A","A","B"),size=c(5.5,5.5,5.5), color='Navy Blue') +annotate("segment", x = 2, xend = 3, y = 16.5, yend = 16.5, size = 0.5, color = "black", linetype = "solid")+annotate("segment", x = 2, xend = 2, y = 16.5, yend = 15.5, size = 0.5)+annotate("segment", x = 3, xend = 3, y = 16.5, yend = 15.5, size = 0.5)+annotate("segment", x = 4, xend = 5, y = 21, yend = 21, size = 0.5, color = "black", linetype = "solid")+annotate("segment", x = 4, xend = 4, y = 21, yend = 20, size = 0.5)+annotate("segment", x = 5, xend = 5, y = 21, yend = 20, size = 0.5)+annotate("text", x = c(2.5,4.5), y = c(18,22.5),label = c("mean: 8.11", "mean: 13.01") , color="black", size=4.5 , angle=0 ,fontface="bold")+annotate("text", x = c(1,2,3,4,5), y = c(14,12.5,14,18.5,18.5),label = c("b","b","b","b","a") , color="black", size=4.5 , angle=0 ,fontface="bold")

**
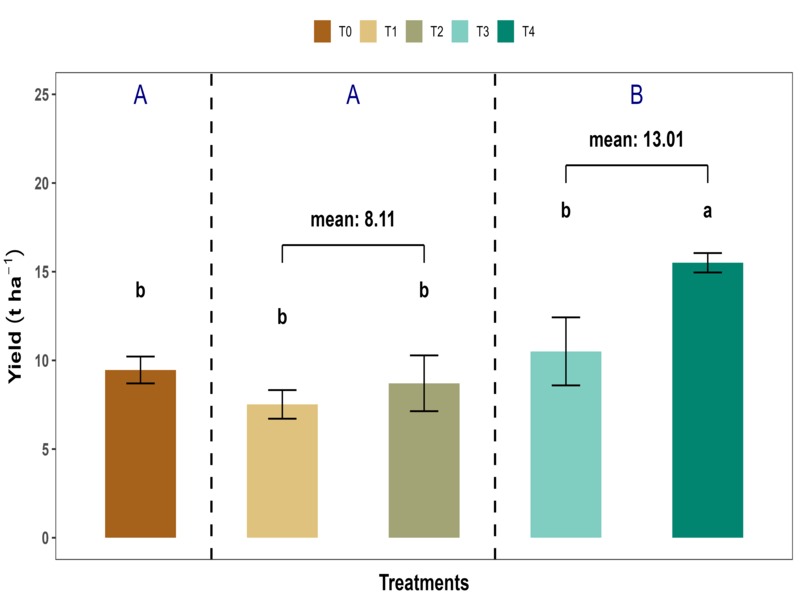
**

**Fig 5. Fruit yield (t ha^-1^) of *Capsicum frutescens* under different fertilization treatments.**

**Vitamin C (a)**

ggplot(tgc, aes(x=T, y=v, color=T))+ theme_bw()+ geom_errorbar(aes(ymin=v-se, ymax= v+se), width=.10, size=1, position=position_dodge(0.8))+ geom_point(position=pd, size=3)+theme_bw()+ theme(axis.text.y = element_text(size=11, face="bold"))+ theme(axis.text.x = element_text(size=11, face="bold"))+ geom_rect(data=NULL,aes(xmin=0.4,xmax=1.5,ymin=-Inf,ymax=Inf),fill="lightgray", alpha=0.1)+ geom_rect(data=NULL,aes(xmin=1.5,xmax=3.5,ymin=-Inf,ymax=Inf),fill="pink", alpha=0.1)+ theme(panel.border = element_rect(size=2))+ theme(panel.grid.major = element_blank(), panel.grid.minor = element_blank())+ labs(y=expression('Vitamin C'~~(mg~100~g^-1)))+ theme(legend.position = 'none')+ ylim (95,130) + annotate("text",x= 1, y = 117, label = "e", size= 4.5)+ annotate("text",x= 2, y = 104, label = "d", size= 4.5) + annotate("text",x= 3, y = 109, label = "c", size= 4.5) + annotate("text",x= 4, y = 122, label = "b", size= 4.5)+ annotate("text",x= 5, y = 128, label = "a", size= 4.5)+ theme(axis.title.x = element_blank())+ theme(axis.title.y = element_text(size=11, face="bold"))

**Capsaicin content (b)**

ggplot(tgc, aes(x=T, y=cp, color=T))+ theme_bw()+ geom_errorbar(aes(ymin=cp-se, ymax= cp+se), width=.10, size=1, position=position_dodge(0.8))+ geom_point(position=pd, size=3)+theme_bw()+ylim (0.87, 1.3) + theme(axis.text.y = element_text(size=11, face="bold"))+ theme(axis.text.x = element_text(size=11, face="bold"))+ geom_rect(data=NULL,aes(xmin=0.4,xmax=1.5,ymin=-Inf,ymax=Inf),fill="lightgray", alpha=0.1)+ geom_rect(data=NULL,aes(xmin=1.5,xmax=3.5,ymin=-Inf,ymax=Inf),fill="pink", alpha=0.1)+ theme(panel.border = element_rect(size=2))+ theme(panel.grid.major = element_blank(), panel.grid.minor = element_blank())+ labs(y=expression('Capsaicin Content (%)'))+ theme(legend.position = 'none')+ annotate("text",x= 1, y = 1.13, label = "e", size= 4.5)+ annotate("text",x= 2, y = 0.95, label = "d", size= 4.5) + annotate("text",x= 3, y = 1.05, label = "c", size= 4.5) + annotate("text",x= 4, y = 1.18, label = "b", size= 4.5)+ annotate("text",x= 5, y = 1.27, label = "a", size= 4.5)+ theme(axis.title.x = element_blank())+ theme(axis.title.y = element_text(size=11, face="bold"))

**SPAD reading (c)**

ggplot(tgc, aes(x=T, y=SP, color=T))+ theme_bw()+ geom_errorbar(aes(ymin=SP-se, ymax= SP+se), width=.10, size=1, position=position_dodge(0.8))+ geom_point(position=pd, size=2)+theme_bw()+ theme(axis.text.y = element_text(size=11, face="bold"))+ theme(axis.text.x = element_text(size=11, face="bold"))+ geom_rect(data=NULL,aes(xmin=0.4,xmax=1.5,ymin=-Inf,ymax=Inf),fill="lightgray", alpha=0.1)+ geom_rect(data=NULL,aes(xmin=1.5,xmax=3.5,ymin=-Inf,ymax=Inf),fill="pink", alpha=0.1)+ theme(panel.border = element_rect(size=2))+ theme(panel.grid.major = element_blank(), panel.grid.minor = element_blank())+ ylab("SPAD Reading")+ theme(legend.position = 'none') + ylim (35, 52) + annotate("text",x= 1, y = 39, label = "e", size= 4.5)+ annotate("text",x= 2, y = 43, label = "d", size= 4.5) + annotate("text",x= 3, y = 45, label = "c", size= 4.5) + annotate("text",x= 4, y = 48, label = "b", size= 4.5)+ annotate("text",x= 5, y = 50, label = "a", size= 4.5)+ theme(axis.title.y = element_text(size=11)) + geom_segment(aes(x=0.6, y=40, xend=1.4, yend=40),colour='red', size=0.8, linetype= "twodash") + annotate(geom = "text", x = 1, y = 41, label = "Control", size=2.8, col='black') + geom_segment(aes(x=1.5, y=48, xend=3.5, yend=48),colour='red', size=0.8, linetype='twodash') + annotate(geom = "text", x = 2.5, y = 49, label = "Organic Fertilizer", size=2.8, col='black')+ geom_segment(aes(x=3.5, y=51, xend=5.5, yend=51),colour='red',size=0.8,linetype='twodash') + annotate(geom = "text", x = 4.5, y = 51.8, label = "Mixed Fertilizer", size=2.8, col='black')+ xlab('Treatments')+ theme(axis.title.x = element_text(size=11, face="bold"))

**
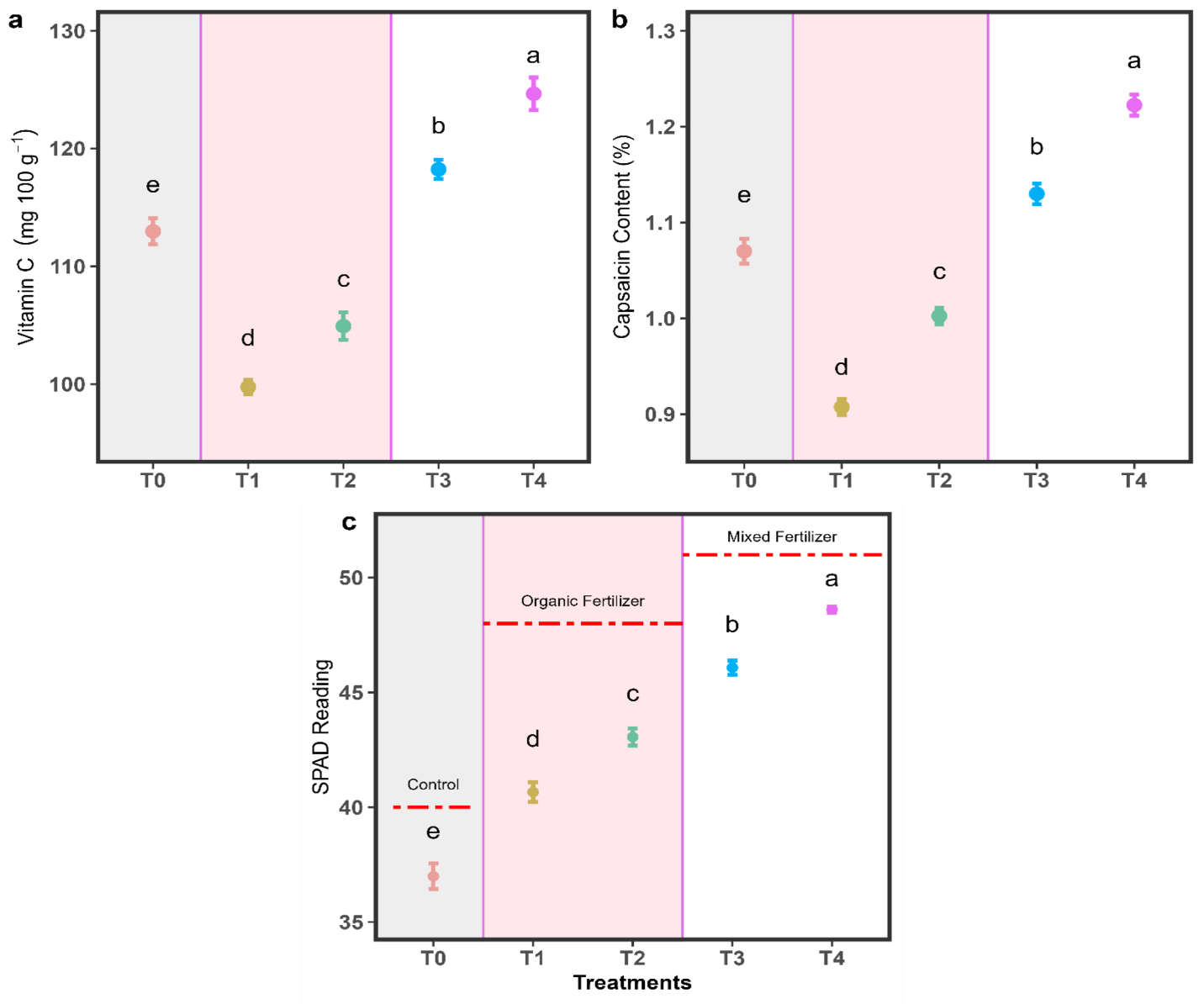
**

**Fig 6. Vitamin C, capsaicin content and SPAD reading of *Capsicum frutescens* under different fertilization treatments.**

**ANPP vs number of leaves (a)**

ggplot(AL, aes(x=Leaf, y=ANPP, colour = T)) + labs(x=expression(bold('Number of leaves'~(plant^-1))))+labs(y=expression(bold('ANPP'~(g~plant^-1))))+geom_point(aes(colour = T), size = 3.5)+ geom_smooth(aes(color=T), method=lm, fullrange=FALSE, se=FALSE)+theme_bw()+ stat_smooth(method = "lm",size = 1, se = TRUE, colour = "black", fill='lightgray', linetype="dashed")+ scale_colour_manual(values = c('coral1','darkgoldenrod2','darkcyan','gray62', 'olivedrab4'))+theme(panel.grid.major = element_blank(), panel.grid.minor = element_blank())+theme(axis.text=element_text(size=10,face='bold'))+theme(axis.title.y = element_text(vjust = +2))+theme(axis.title.x = element_text(vjust = 0))+ theme(legend.position=c(0.12,0.83))+ guides(colour = guide_legend( label.position = "left"))+ theme(legend.key.width=unit(0.25,"cm"),legend.key.height=unit(0.48,"cm"))+theme(legend.title = element_blank())+ theme(legend.text = element_text(size= 8.5))+ylim(0,40)+theme(axis.title = element_text(face="bold", size=13)) +annotate("text", x = c(355,355,355,355,355,355), y = c(38.6,36.5,34.5,32.5,30.5,28.5), label = "italic(R)^2", parse = TRUE ,size=3.5,color=c("coral1","darkgoldenrod2","darkcyan","gray62","olivedrab4","black")) +annotate("text", x = c(405,405,405,405,405,405), y = c(38.6,36.5,34.5,32.5,30.5,28.5), label = c(" = 0.44 ,", " = 0.47 ,"," = 0.51 ,"," = 0.55 ,"," = 0.57 ,"," = 0.51 ,") , color=c("coral1","darkgoldenrod2","darkcyan","gray62","olivedrab4","black"), size=3.5 , angle=0)+annotate("text", x = c(460,460,460,460,460,460), y = c(38.6,36.5,34.5,32.5,30.5,28.5), label = "p" , color=c("coral1","darkgoldenrod2","darkcyan","gray62","olivedrab4","black"), size=3.5 , angle=0,fontface="italic")+annotate("text", x = c(505,505,505,505,505,505), y = c(38.6,36.5,34.5,32.5,30.5,28.5), label = c(" = 0.034", " = 0.030"," = 0.002"," = 0.013"," = 0.006"," = 0.020") , color=c("coral1","darkgoldenrod2","darkcyan","gray62","olivedrab4","black"), size=3.5 , angle=0)

**ANPP vs number of flowering (b)**

ggplot(AF, aes(x = Flowering, y = ANPP, colour = T))+ labs(x=expression(bold('Number of flowering'~(plant^-1))))+labs(y=expression(bold('ANPP'~(g~plant^-1))))+geom_point(aes(colour = T), size = 4)+ geom_smooth(aes(color=T), method=lm, fullrange=FALSE, se=FALSE) +theme_bw()+ stat_smooth(method = "lm",size = 1, se = TRUE, colour = "black", fill='lightgray', linetype="dashed")+ scale_colour_manual(values = c('coral1','darkgoldenrod2','darkcyan','gray62', 'olivedrab4'))+theme(panel.grid.major = element_blank(), panel.grid.minor = element_blank())+theme(axis.text=element_text(size=10,face='bold'))+theme(axis.title.y = element_text(vjust = +2))+theme(axis.title.x = element_text(vjust = 0))+ theme(legend.position=c(0.08,0.85))+ theme(legend.key.width=unit(0.25,"cm"),legend.key.height=unit(0.25,"cm"))+theme(legend.title = element_blank())+ theme(legend.text = element_text(size= 8.5))+ylim(0,40)+theme(axis.title = element_text(face="bold", size=13))+annotate("text", x = c(11.5,11.5,11.5,11.5,11.5,11.5), y = c(38.6,36.5,34.5,32.5,30.5,28.5), label = "italic(R)^2", parse = TRUE,size=3.5,color=c("coral1","darkgoldenrod2","darkcyan","gray62","olivedrab4","black")) +theme(legend.position = "none")+annotate("text", x = c(14.8,14.8,14.8,14.8,14.8,14.8), y = c(38.6,36.5,34.5,32.5,30.5,28.5), label = c(" = 0.38 ,", " = 0.06 ,"," = 0.67 ,"," = 0.27 ,"," = 0.68 ,"," = 0.41 ,") , color=c("coral1","darkgoldenrod2","darkcyan","gray62","olivedrab4","black"), size=3.5 , angle=0)+annotate("text", x = c(18.3,18.3,18.3,18.3,18.3,18.3), y = c(38.6,36.5,34.5,32.5,30.5,28.5), label = "p" , color=c("coral1","darkgoldenrod2","darkcyan","gray62","olivedrab4","black"), size=3.5 , angle=0,fontface="italic")+annotate("text", x = c(20.9,20.9,21.2,20.9,21.2,21.2), y = c(38.6,36.5,34.5,32.5,30.5,28.5), label = c(" = 0.18", " = 0.75"," < 0.001"," = 0.22"," < 0.001"," = 0.014") , color=c("coral1","darkgoldenrod2","darkcyan","gray62","olivedrab4","black"), size=3.5 , angle=0)

**
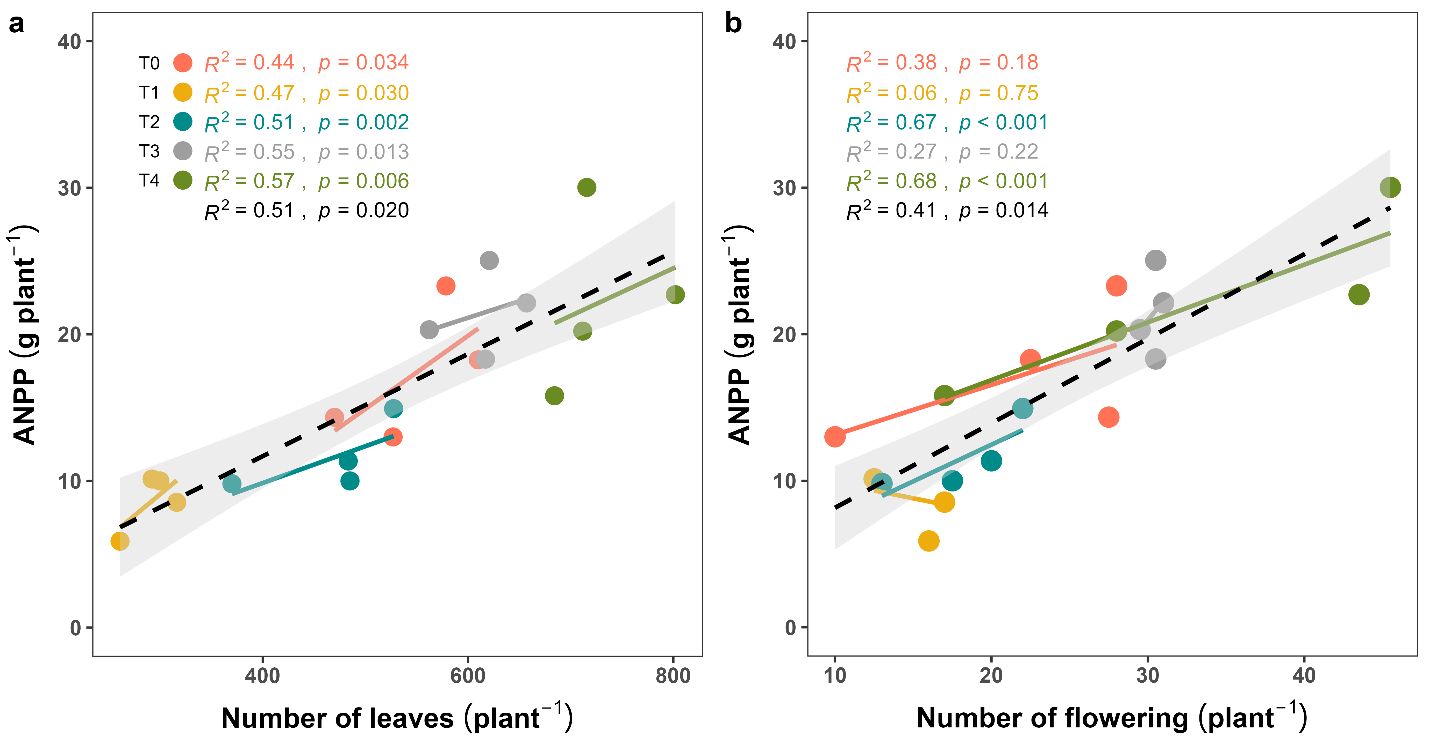
**

**Fig 7. Regression analysis between ANPP and vegetative (leaf number) and reproductive (flower number) traits of *Capsicum frutescence.***

**Yield vs number of leaves (a)**

ggplot(YL, aes(x = Leaf, y = Yield, colour = T)) + labs(x=expression(bold('Number of leaves'~(plant^-1))))+labs(y=expression(bold('Yield'~(t~ha^-1))))+geom_point(aes(colour = T), size = 3.5)+ geom_smooth(aes(color=T), method=lm, fullrange=FALSE, se=FALSE)+theme_bw()+ stat_smooth(method = "lm",size = 1, se = TRUE, colour = "black", fill='lightgray', linetype="dashed")+ scale_colour_manual(values = c('coral1','darkgoldenrod2','darkcyan','gray62', 'olivedrab4'))+theme(panel.grid.major = element_blank(), panel.grid.minor = element_blank())+theme(axis.text=element_text(size=10,face='bold'))+theme(axis.title.y = element_text(vjust = +2))+theme(axis.title.x = element_text(vjust = 0))+ theme(legend.position=c(0.12,0.83))+ guides(colour = guide_legend( label.position = "left"))+ theme(legend.key.width=unit(0.25,"cm"),legend.key.height=unit(0.48,"cm"))+theme(legend.title = element_blank())+ theme(legend.text = element_text(size= 8.5))+ylim(0,30)+theme(axis.title = element_text(face="bold", size=12)) + annotate("text", x = c(355,355,355,355,355,355), y = c(28.9,27.4,25.9,24.4,22.9,21.4), label = "italic(R)^2", parse = TRUE,size=3.5,color=c("coral1","darkgoldenrod2","darkcyan","gray62","olivedrab4","black"))+annotate("text", x = c(405,405,405,410,405,405), y = c(28.9,27.4,25.9,24.4,22.9,21.4), label = c(" = 0.32 ,", " = 0.40 ,"," = 0.80 ,"," = 0.041 ,"," = 0.12 ,"," = 0.34 ,") , color=c("coral1","darkgoldenrod2","darkcyan","gray62","olivedrab4","black"), size=3.5 , angle=0)+annotate("text", x = c(460,460,460,470,460,460), y = c(28.9,27.4,25.9,24.4,22.9,21.4), label = "p" , color=c("coral1","darkgoldenrod2","darkcyan","gray62","olivedrab4","black"), size=3.5 , angle=0,fontface="italic")+annotate("text", x = c(500,504,503,503,498,511), y = c(28.9,27.4,25.9,24.4,22.9,21.4), label = c(" = 0.33", " = 0.041"," < 0.001"," = 0.8"," = 0.65"," = 0.002") , color=c("coral1","darkgoldenrod2","darkcyan","gray62","olivedrab4","black"), size=3.5 , angle=0)

**Yield vs number of flowers (b)**

ggplot(YF, aes(x = Flowering, y = Yield, colour = T)) + labs(x=expression(bold('Number of flowering'~(plant^-1))))+labs(y=expression(bold('Yield'~(t~ha^-1))))+geom_point(aes(colour = T), size = 3.5)+ geom_smooth(aes(color=T), method=lm, fullrange=FALSE, se=FALSE)+theme_bw()+ stat_smooth(method = "lm",size = 1, se = TRUE, colour = "black", fill='lightgray', linetype="dashed")+ scale_colour_manual(values = c('coral1','darkgoldenrod2','darkcyan','gray62', 'olivedrab4'))+theme(panel.grid.major = element_blank(), panel.grid.minor = element_blank())+theme(axis.text=element_text(size=10,face='bold'))+theme(axis.title.y = element_text(vjust = +2))+theme(axis.title.x = element_text(vjust = 0))+ theme(legend.position=c(0.12,0.83))+ guides(colour = guide_legend( label.position = "left"))+ theme(legend.key.width=unit(0.25,"cm"),legend.key.height=unit(0.48,"cm"))+theme(legend.title = element_blank())+ theme(legend.text = element_text(size= 8.5))+ylim(0,30)+theme(axis.title = element_text(face="bold", size=12)) +theme(legend.position = "none")+annotate("text", x = c(11.5,11.5,11.5,11.5,11.5,11.5), y = c(28.9,27.4,25.9,24.4,22.9,21.4), label = "italic(R)^2", parse = TRUE,size=3.5,color=c("coral1","darkgoldenrod2","darkcyan","gray62","olivedrab4","black"))+annotate("text", x = c(14.8,14.8,14.8,14.8,14.8,14.8), y = c(28.9,27.4,25.9,24.4,22.9,21.4), label = c(" = 0.27 ,", " = 0.33 ,"," = 0.82 ,"," = 0.40 ,"," = 0.28 ,"," = 0.42 ,") , color=c("coral1","darkgoldenrod2","darkcyan","gray62","olivedrab4","black"), size=3.5 , angle=0)+annotate("text", x = c(18.3,18.3,18.3,18.3,18.3,18.3), y = c(28.9,27.4,25.9,24.4,23.0,21.4), label = "p" , color=c("coral1","darkgoldenrod2","darkcyan","gray62","olivedrab4","black"), size=3.5 , angle=0,fontface="italic")+annotate("text", x = c(20.9,21.3,21.2,21.3,20.9,21.3), y = c(28.9,27.4,25.9,24.4,22.9,21.4), label = c(" = 0.39", " = 0.046"," < 0.001"," = 0.031"," = 0.41"," = 0.037") , color=c("coral1","darkgoldenrod2","darkcyan","gray62","olivedrab4","black"), size=3.5 , angle=0)

**
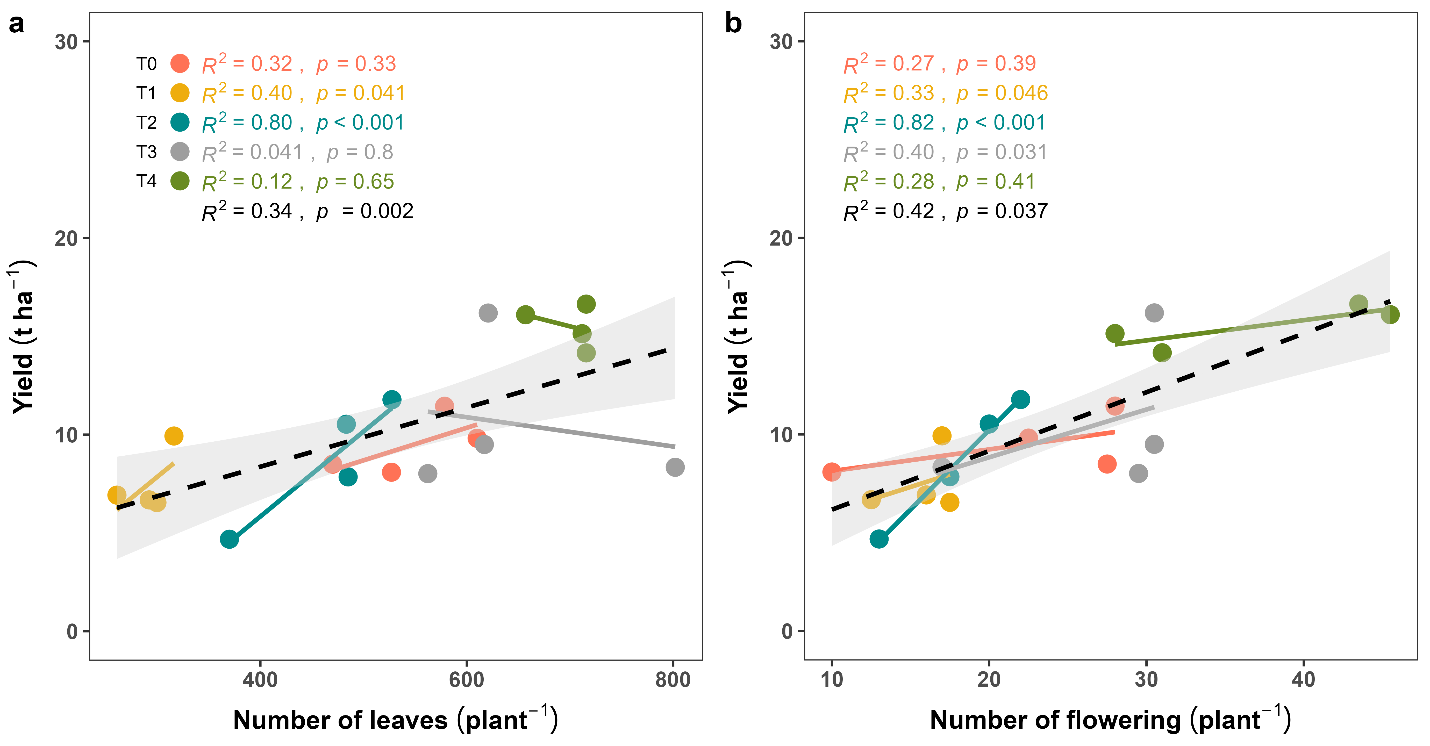
**

**Fig 8. Regression analysis showing relationship between fruit yield and vegetative-reproductive traits (leaf and flower number) in *Capsicum frutescence.***
