# Supplementary material for: Beyond mono fertilization: Mixed fertilization enhances productivity and quality of chili (Capsicum frutescens)
Source: PLoS One. 2026 Mar 31;21(3):e0331538. doi: 10.1371/journal.pone.0331538 (PMC13037990; doi:10.1371/journal.pone.0331538)
Supplement: Attachment — Submitted filename: Response to Reviewers.docx [file pone.0331538.s004.docx]

**Response to Reviewer # 1**

**Reviewer Comment**

The title is confusing. I recommend rewriting it. The short title works better and should be used.

**Author Response**

We thank the reviewer for this valuable comment. Following reviewer suggestion, we have rewritten the title to make it concise and clear. The revised title is:

*“****Beyond mono fertilization: Mixed fertilization enhances productivity and quality of chili (Capsicum frutescens)****”*

**Reviewer Comment**

The first two keywords are suitable. NPP is ambiguous; use “net primary productivity.” “Mixed fertilization” is uncommon and may reduce visibility. “Quality” and “yield” are too broad. I suggest keywords like “integrated nutrient management” or “plant nutrition.”

**Author Response**

Thank you for the detailed guidance. We have revised the keywords following your advice. The revised set of keywords is:

***Chili, Leucaena leucocephala, net primary productivity, integrated nutrient management***

**Reviewer Comment**

If the authors used “recommended doses of fertilizers,” the acronym “RDF” suits better than “RFD,” and they will need to replace RFD with RDF. If they write “recommend fertilizer doses,” they can keep “RFD.” Both formats are appropriate, but the authors must be consistent in the whole document and write only “recommended doses of fertilizers (RDF)” or “recommended fertilizer doses (RFD).”

**Author Response**

*We thank the reviewer for this important clarification. We have carefully checked the manuscript and maintained consistency throughout the manuscript. The term “recommended doses of fertilizers (RDF)” has been adopted and uniformly applied across the abstract and the entire manuscript to ensure accuracy and standard usage.*

**Reviewer Comment**

The abstract has much data and results. Please summarize the information and minimize the amount of numbers, emphasizing the trends.

**Author Response**

Thank you for this helpful suggestion. We have rewritten the abstract to improve readability and focus on the main outcomes rather than detailed statistics. The revised version emphasizes the overall trend-that combining *Leucaena* *leucocephala* leaf litter with inorganic fertilizers enhanced chili growth, yield and quality compared to single fertilizer use. We believe our revised abstract will be easily understandable for the reader. Our revised abstract is now like “*While the effects of different fertilization strategies on chili (Capsicum frutescens) cultivation have been previously examined, the comparative assessment of mixed versus mono fertilization approaches limit our understanding. To address this gap, we have conducted an experiment using a completely randomized design (CRD) with five fertilization treatments along with four replications of each treatment (e.g., Leucaena leucocephala leaf litter and recommended dose of synthetic fertilizers (RDF)) to observed the effect of these treatments on plant productivity traits, NPP (net primary productivity), quality and overall yield of chili. We found that mixed fertilization (T₄: 24.8 g kg⁻¹ leaf litter + ½ RDF) results higher yield (15.5 t ha^-1^), which is about 64% and 92% greater than control (T_0_) and organic fertilization (T_1_ + T_2_) respectively. The T_4_ treatment, showed consistently higher plant height, leaf and fruit number at 60 DAT, as well as higher flowering at 45 DAT compared to other treatments. Net primary productivity (above- and belowground combined) was also higher in T_3_ and T₄ treatments (26.47 g plant^-1^) compared to control and organic fertilization. Similarly, quality traits such as vitamin C (124.65 mg 100 g^-1^), capsaicin content (1.22%) and SPAD reading (48.61) were significantly increased under mixed fertilization (with in each treatment: p < 0.001). In addition linear fit regression analysis indicated that both ANPP and yield were significantly related with leaf number (ANPP: p = 0.02; Yield: p = 0.002) and flowering (ANPP: p = 0.014; Yield: p = 0.037). Our results further suggest that mixed fertilization enhances both productivity and quality traits of chili, therefore, promotion of integrated (organic and inorganic together) fertilizer management practices is recommended to improve yield and quality of chili.”*

**Reviewer Comment**

The flow of the Introduction needs improvement. The authors mix and split ideas across paragraphs and include too many examples. Please rewrite the Introduction and consider a clear order of topics. I suggest, as an example, the following flow of paragraphs:

The economic importance of chili and the need to improve the quantity and quality of the crops > Importance of fertilization for crops, especially chili, with examples of inorganic and organic fertilizers, especially lead tree leaf litter > Explanation of the advantages and disadvantages of organic and inorganic fertilizers, and the advantages of mixing them both > Hypothesis and objective of this work.

Please remember not to cite more than two approaches with inorganic fertilization, two approaches with organic fertilization, and two mixed fertilization approaches. The authors must emphasize their research, not make an extensive literature review. This is a research article, not a review article.

**Author Response**

*We appreciate the reviewer’s thoughtful and detailed guidance. The Introduction has been completely reorganized and rewritten with appropriate logical flow as suggested. Redundant examples and excessive citations have been removed to focus on the study’s rationale and objectives.*

*The revised version now follows the structure recommended by the reviewer:*

*> We added and revised the economic importance of chili as suggested (please see L 44-51).*

*> We incorporate the role of fertilizers on chili production and sufficiently introduce the influence of both organic and inorganic fertilizer on growth and quality traits of chili, (please see L 51-53).*

*> We carefully and logically added the advantages and limitations of using organic or inorganic fertilizers following close related review (please see L 53-65).*

*> We revised the introduction part of our manuscript, regarding integrated Leucaena leucocephala leaf litter with inorganic fertilizers as a mixed fertilization strategy (please see L 65-82).*

*> Following reviewer suggestion we also added the hypothesis of our experiment (please see L 87-89).*

*We kept closely related review as citation in the introduction section as well as throughout the manuscript and additional citations are removed, as advised (Excluded those citations such as 9, 10,11,12,15,20,21,22,23,26,27,29,30,32,33)*

**Reviewer Comment**

2.1. Experimental site and plant material: OK

**Author Response**

*We appreciate your comments. We have made no changes as suggested.*

**Reviewer Comment**

2.2. Pot preparation and experimental design-Figure 2 is good, but if the authors could also add a real picture of just the pots, as an example, the engagement with readers may increase. If the authors have no picture of the pots used in this experiment, please disregard this comment.

**Author Response**

*Thank you for your comment. Our schematic representation of the field layout (figure 2) clearly represented our experiment along with exact experimental design. We design it such a way that reader can easily understand. We also have the real pictures of our field layout, however, those pictures are not properly organized to put it in the main manuscript. For your kind concern we have added some pictures below.*

**Reviewer Comment**

2.2.1. Fertilizer application-There are flaws in the experimental design. The authors have two factors (organic and inorganic fertilizers) in three levels (none, half, full). The correct experimental design should be 3² = 9 treatments, not only five. The missing treatments are 0 organic + 0 inorganic; 0 organic + 0.5 inorganic; 0.5 organic + 1 inorganic; and 1 organic + 1 inorganic. It is too late now to repeat the experiment with the missing treatments. Therefore, the authors must acknowledge the incomplete set of treatments as a weak point of this document, which should be addressed in future works, and provide an explanation for the incomplete set. This explanation could range from a lack of enough pots to a scientific reason that the missing treatments are unrealistic for chili production. Please look for statistical support for further work to avoid missing treatments again.

**Author Response**

*We thank the reviewer for the valuable comment. As we follow the standard procedure of chili cultivation given by Bangladesh Agricultural Research Institute (BARI) for the chili variety (BARI- 3), we incorporated all possible treatment accordingly. Although some other fertilization treatment (none, half and full), as you suggested could improve our experiment, unfortunately we did not consider it at the begging of our experiment and we agree that it is our limitation. As you advice we added this gap as a limitation of our study and suggested for the future experiment.*

**Reviewer Comment**

Table 2: Did the authors apply two doses of gypsum to the pots? In the ½ dose, they write in the caption “Urea (0.50 g),…Gypsum (0.30 g), Boric acid (0.13 g), Gypsum (0.13 g)…”. Please recheck and fix the caption, if needed.

**Author Response**

*We thank the reviewer for noticing this inconsistency. The duplication of “Gypsum” in the caption was a typographical error. It has now been corrected, and the fertilizer composition table has been reviewed thoroughly for accuracy (See Table 2, Line 161).*

**Reviewer Comment**

2.2.2. Application of litterbag technique-Please do not provide results in the Materials and Methods. Keep all relevant results for the Results or Discussion sections. Therefore, remove the excerpt “Chemical analysis … nutrient release.”

**Author Response**

*We agree with the reviewer’s observation. The sentence containing partial results (“Chemical analysis of the decomposed litter and surrounding soil revealed significant nutrient enrichment. The results showed a measurable release of key nutrients (Table 2). These findings highlight the role of L. leucocephala litter in enhancing soil fertility and supporting plant growth through sustained nutrient release”) has been deleted from the Materials and Methods* 2.2.2 *section. All relevant information now appears appropriately in the results section (please see L 164-171in the revised manuscript).*

**Reviewer Comment**

2.3. Data collection-Please define the acronym BNPP the first time it is used.

**Author Response**

*We appreciate this reminder. The acronym* ***BNPP (Belowground Net Primary Productivity)*** *is now defined at its first appearance in the Data Collection section (please see L 184).*

**Reviewer Comment**

2.4. Data analysis: OK

**Author Response**

*We appreciate the reviewer’s confirmation.*

**Reviewer Comment**

3.1 Mixed fertilization significantly enhanced growth dynamics- The claim “At 15 DAT, differences among treatments were minimal (Figs 3a and 4a)” is not right. There is no statistical difference for plant height at 15 DAT across the treatments, but there is a difference for the number of leaves. Please rewrite this result.

**Author Response**

*Thanks for the reviewer comment. Following the suggestion, we have rewritten section 3.1 to clearly state that plant height (Fig 3a) showed no significant difference at 15 DAT, whereas leaf number (Fig 3b) varied significantly among treatments. Additionally, we have combined all four parameters (plant height, leaf number, flowering number and fruit number) into a single, comprehensive Figure 3 (previously presented in figures 3,4,5,6) for improved clarity and comparison (please see L 233-249).*

**Reviewer Comment**

“Figure 3: Please check the letters for treatments. It does not make sense that the ‘ab’ treatment is greater than ‘b’ and smaller than ‘c’, without any ‘a’ treatment in the graph.

**Author Response**

*We appreciate the reviewer’s observation. We have revised and reproduced some figures in our manuscript showing with and between treatment effect on vegetative and reproductive trait of chili, using ANOVA and Tukey’s HSD test (p < 0.05) to ensure accurate statistical grouping. The letters in the revised Figure 3a have been corrected to follow the standard logical order (a > b > c).*

**Reviewer Comment**

3.2 Enhanced reproductive traits under mixed fertilization- Figure 6: Please check the letters for treatments. It does not make sense that the ‘ab’ treatment is greater than ‘b’ and smaller than ‘c’, without any ‘a’ treatment in the graph.

**Author Response**

*We rechecked and corrected the statistical letters in Figure 3d using Tukey’s HSD analysis. The labeling sequence has been reordered consistently (a > b > c) to accurately represent treatment differences. Please see the updated Figure 3d (previously presented in figure 6).*

**Reviewer Comment**

3.3 Net primary productivity maximized under mixed inputs- Provide the information on the meaning of ‘a’ (Aboveground net primary productivity-ANPP), ‘b’ (Belowground net primary productivity-BNPP), and ‘c’ (Total net primary productivity-ANPP + BNPP). Additionally, the letters within graphs are weird. Figure 7a has ‘b’ for the smaller values, ‘c’ for the highest values, and ‘a’ for the intermediate values. This is out of the regular order… Please redo the lettering.

**Author Response**

*Thank you for your suggestion. The caption of figure 7 (now presented in figure 4) has been revised as follows: “(a) Aboveground Net Primary Productivity (ANPP) and (b) Belowground Net Primary Productivity (BNPP) (L 263-265) of Capsicum frutescens under different fertilization treatments.” We also corrected the lettering order in all plots to follow the conventional statistical hierarchy (a > b > c). Please see the corrected Figure 4.*

**Reviewer Comment**

3.4 Mixed fertilization significantly boosted fruit yield - I suggest moving the discussion to the ‘Discussion’ section… the authors claim that T₃ is ‘slightly’ lower than T₄… This difference seems to be significant, not slight. Please redo the discussion when moving to another section.”

**Author Response**

*We agree with the reviewer’s observation. We have removed the word “slightly” and replaced it with “significant difference” to accurately describe the statistical difference between T₃ and T₄ (p < 0.05) (please see L 272-274). The relevant portion of Section 3.4 (now presented 3.3) has been rewritten and removed discussion related sentences from the result section (please see track file L 370, 372-374, 376, 377).*

**Reviewer Comment**

3.5 Please adjust the bars to not begin from 0. The current format does not allow to make the differences visually remarkable. Furthermore, please add the letters for the statistical analysis.

**Author Response**

*Thanks for your comments, we revised the figures and y axis are now not began from 0. We also added statistical letters derived from ANOVA and Tukey’s HSD at (p < 0.05) in each plot (vitamin C, capsaicin content, and SPAD reading). Please see the corrected figure 6 (previously presented in figure 9).*

**Reviewer Comment**

3.6 Significant associations between ANPP and vegetative reproductive traits: OK

**Author Response**

*Thank you for your comments.*

**Reviewer Comment**

3.7 Yield strongly influenced by vegetative and reproductive structures: OK

**Author Response**

*We appreciate the reviewer’s confirmation.*

**Reviewer Comment**

4.1 Effect of mixed and mono fertilization on yield-contributing traits of chili- The claim that “…mixed fertilization (T₄) consistently outperforming mono strategies at all observation stages” is right only for the number of leaves, not for all vegetative traits. Please redo the discussion.

**Author Response**

*We apologized for our mistake. In our experiment mixed fertilization (T₄) alone did not consistently outperform all mono strategies across every vegetative trait. However, both mixed fertilization treatments (T₃ + T₄) showed significant differences compared with the control and organic fertilizer treatments for plant height and leaf number (please see L 315-316). The discussion has been revised accordingly to reflect these results more accurately.*

**Reviewer Comment**

4.2 Effect of mixed and mono fertilization on NPP and fruit quality- The results are difficult to follow because there is no statistical analysis for the individual treatments. The authors merged T_1_/T_2_ and T_3_/T_4_, making the individual comparison between T₄ and other treatments more complex. The authors can keep their current analysis of figures 7 and 8, but they must also present the statistical analysis for individual treatments.

**Author Response**

*We thank for your comment regarding our statistical comparison test. In the revised manuscript, we have maintained the grouped comparisons (T_1_ + T_2_ as organic and T_3_ + T_4_ as mixed fertilizers). We revised and represent the statistical analysis for each individual treatment in figure 4 and figure 5 (previously figure 7 and 8 respectively).*

**Reviewer Comment**

4.3 Relationships among morphological traits, biomass accumulation and fruit yield: *OK*

**Author Response**

*We appreciate your comment.*

**Reviewer Comment**

5 Conclusions: OK

**Author Response**

*Thanks.*

**Reviewer Comment**

**Acknowledgments, **Author Contribution, **Competing Interests: OK

**Author Response**

*Thanks.*

**Reviewer Comment**

References- Please be consistent with the titles of the articles. Either capitalize only the first word at the beginning of the title or capitalize the first word and all nouns. Please choose one format and write all the references in this format, even if the articles were published otherwise.

**Author Response**

*We sincerely appreciate this valuable suggestion. All references have been standardized to follow a single format where capitalize only the first word at the beginning of the* *title, in accordance with PLOS ONE style using reference managing software ‘ZOTERO”. Example of applied format: “Alleviation of fungicide-induced phytotoxicity in greengram [Vigna radiata (L.) Wilczek] using fungicide-tolerant and plant growth-promoting Pseudomonas strain.” All reference titles were revised consistently throughout the reference list (revised reference no. 1,2,7,10,11,12,13,14,15,16,20,22,23,28,33,34,46,55,56).*

**Response to Reviewer # 2**

**Reviewer Comment**

Each treatment was designed to include four replicates. Although according to the “vegetable research method”, at least three biological replicates are required, as a field application-oriented fertilizer comparison test, only four seedlings were designed for each treatment, which is a relatively small number.

**Author Response**

*We thank the reviewer for this valuable observation. In our study, we used four biological replicates per treatment following a Completely Randomized Design (CRD), where each replicate consisted of a single plant. The experiment was conducted under controlled outdoor conditions using ideal plastic pots (30 cm height × 28 cm diameter) developed by Rangpur Foundry Limited (RFL) Company, Bangladesh, specifically designed for crop production. Initially, we transplanted two seedlings per pot to monitor early growth performance. However, we later removed one seedling from each pot and allowed a single seedling to grow. This decision was made because the pot size was not suitable for two seedlings due to spatial limitations, which could lead to above- and belowground competition. Maintaining one seedling per pot also ensured accurate assessment of nutrient utilization under each treatment (please see L 132-134). Moreover, we followed previous established experimental design for crop cultivation in pot based experiment, where one seedling per pot was used for uniform plant growth under reliable imposed treatment (Ij et al., 2022; Kajeevan & Gnanavelrajah, 2023; Chukwuka et al., 2014) (please see L 193-200).*

**Reviewer Comment**

It should be clearly stated whether the experiment was conducted in a greenhouse or outdoors. Figure 1 is meteorological data and is not directly related to the research content. Therefore, Figure 1 can be deleted.

**Author Response**

*We thankful to the reviewer for this observation. The experiment was conducted outdoors at the Agroforestry research farm of Sher-e-Bangla Agricultural University under open-field but controlled pot conditions. Regarding figure 1, we respectful to the reviewer but we want to keep it because the meteorological data derived from the meteorological station- Bangladesh Meteorological Department (BMD), located very close to our experimental field, which provide essential background for interpreting plant growth and productivity responses. Since the experiment was conducted outdoors, we observed variations in temperature, rainfall and humidity during the whole experimental period and we assume those have influence on chili cultivation. We strongly believe that those meteorological data will helps the readers to understand the treatment effect in respect to local environmental context. We clarified this in the revised text. (Please see L 95-105).*

**Reviewer Comment**

The plant heights in Figure 3 can be edited into a bar chart. Similarly, the number of leaves in Figure 4, the number of flowers in Figure 5, the number of fruits in Figure 6, and the yield per plant in Figure 7 can all be summarized. Each indicator can be edited into a bar chart and then all combined into one figure.

**Author Response**

*We appreciate the reviewer’s valuable suggestion to improve figure clarity and presentation. Following reviewer suggestion, we have combined the growth parameters- plant height, leaf number, flower number and fruit number- into a single composite bar chart, now presented as figure 3 (previously presented in figure 3, 4, 5 and 6) to ensure each parameter is clearly visible and easy to interpret. Regarding yield, we would like to clarify that figure 7 (now presented figure 4) represents net primary productivity (NPP), while yield (expressed as t/ha) is shown in Figure 8 (now presented figure 5), following standard agronomic reporting conventions (please see L 414-416). This value was derived from per-plant yield and then converted to a hectare basis using the standard conversion formula cited in the methods (*[*www.agriculture.vic.gov.au*](http://www.agriculture.vic.gov.au)*) and also cross checked by Gomez & Gomez [33] (Please see L 193-200). Our revised figure related to yield is now presented in figure 5 as a bar chart. Figure 4 (NPP) has been kept separate as it represents biomass productivity rather than yield.*

**Reviewer Comment**

Similarly, the content of Vitamin C can be summarized into a bar chart. The effects of fertilizers on appearance indicators and internal quality can be divided, with appearance indicators combined in one large chart and internal quality in another.

**Author Response**

*We thank the reviewer for valuable suggestion regarding figure organization. Following the recommendation, we have grouped all external (appearance) indicators such as, plant height, leaf number, flower number and fruit number-into a single combined bar chart, now presented as Figure 3 (previously presented in figure 3, 4, 5 and 6) (L 372-410). However, yield was retained separately as Figure 5 (previously presented in figure 8) (L 372-410), since it represents a productivity parameter measured in ton per hectare (t ha⁻¹), which largely differs in scale. For the internal quality indicators, we initially attempted to combine Vitamin C, capsaicin content and SPAD reading into one figure as suggested. However, we found that the capsaicin values were comparatively much lower, making the combined graph visually misleading-its values appeared negligible relative to Vitamin C and SPAD readings and the variation among treatments became unclear. To properly interpretability and ensure accuracy of representation of each parameter, we therefore kept Vitamin C, capsaicin and SPAD reading as three separate line graphs, presented individually in Figures 6 (previously presented in figure 9) (L 372-410). Each figure includes independent statistical analysis (ANOVA and Tukey’s HSD at 0.001 % level of provability), allowing a clear comparison among treatments for each internal quality trait.*

**Reviewer Comment**

This study only has four seedlings, making the calculation of yield per hectare unreliable.

**Author Response**

*We thank the reviewer for this valuable comment. In our experiment, each treatment consisted of four biological replicates, and each replicate bear a single plant grown in each plastic pot (30 cm in height × 28 cm in diameter) (please see L 116-120). For estimating yield (t ha^-1^) we follow the standard procedure and guideline to measure the yield of horticultural crops given by (*[*www.agriculture.vic.gov.au*](http://www.agriculture.vic.gov.au)*). After that we carefully cross checked our yield estimation according to the guideline given by Gomez & Gomez [33] and the procedure of estimating the yield of horticultural crops given by Bangladesh Agricultural Research Institute (*[*www.bari.gov.bd*](http://www.bari.gov.bd)*) to minimize any error of estimation. (Please see L 193-200).*

**Reviewer Comment**

The writing in the abstract is disorganized and requires simplification.

**Author Response**

*We thanks the reviewer for this comment. Following your comment we revised and reorganized our abstract. We also simplified our abstract according to your suggestion so that it is easily understandable for the readers. Our abstract now is like “While the effects of different fertilization strategies on chili (Capsicum frutescens) cultivation have been previously examined, the comparative assessment of mixed versus mono fertilization approaches limit our understanding. To address this gap, we have conducted an experiment using a completely randomized design (CRD) with five fertilization treatments along with four replications of each treatment (e.g., Leucaena leucocephala leaf litter and recommended dose of synthetic fertilizers (RDF)) to observed the effect of these treatments on plant productivity traits, NPP (net primary productivity), quality and overall yield of chili. We found that mixed fertilization (T₄: 24.8 g kg⁻¹ leaf litter + ½ RDF) results higher yield (15.5 t ha^-1^), which is about 64% and 92% greater than control (T_0_) and organic fertilization (T_1_ + T_2_) respectively. The T_4_ treatment, showed consistently higher plant height, leaf and fruit number at 60 DAT, as well as higher flowering at 45 DAT compared to other treatments. Net primary productivity (above- and belowground combined) was also higher in T_3_ and T₄ treatments (26.47 g plant^-1^) compared to control and organic fertilization. Similarly, quality traits such as vitamin C (124.65 mg 100 g^-1^), capsaicin content (1.22%) and SPAD reading (48.61) were significantly increased under mixed fertilization (with in each treatment: p < 0.001). In addition linear fit regression analysis indicated that both ANPP and yield were significantly related with leaf number (ANPP: p = 0.02; Yield: p = 0.002) and flowering (ANPP: p = 0.014; Yield: p = 0.037). Our results further suggest that mixed fertilization enhances both productivity and quality traits of chili, therefore, promotion of integrated (organic and inorganic together) fertilizer management practices is recommended to improve yield and quality of chili.”*

**Reviewer Comment**

There is still a lack of depth in the research content.

**Author Response**

***Thank you for comment. We sufficiently and carefully revised our whole manuscript (abstract, introduction, materials and methods, results, discussion and conclusions).*** *We tried to include all required research content in each parts of our manuscripts. We maintained the appropriate logical flow in the introduction (please see L 43-92), include all required materials following appropriate methods in materials and method section (please see L 93-229). Additionally, we carefully revised, analyzed and prepared graphs following each reviewer comments presented in the result section (please see L 230-308). Finally, we justify and logically interpret our results in discussion section (please see L 309-408). Based on our revised abstract (please see L 20-42), we carefully revised our conclusions too (please see L 409-429). We believe our revised manuscript will sufficiently addressed all suggestions given by the reviewers to improve the quality of our manuscript.*

**Response to Reviewer # 3**

**Reviewer Comment**

Mixed fertilization implies inorganic and organic components in equal or nearly equal proportions. In this case organic manure is only one while inorganic components are six. I think this overshadows the functioning of organic manure. Role of each inorganic fertilizer needs to be given. Role of *L. leucocephala* leaf litter to enhance inorganically fertilized chili production can be included in the title instead of mixed and mono.

**Author Response**

*We appreciate the reviewer’s observation. In our study, the term “mixed fertilization” does not refer to applying organic and inorganic inputs in equal proportions, but to combine one organic source (Leucaena leucocephala leaf litter) with several inorganic fertilizers that supply the essential macro- and micronutrients required for chili production. This approach is consistent with many previous studies where a single organic amendment was used alongside multiple inorganic fertilizers to evaluate integrated nutrient management effects on crop growth and soil health (Malik et al., 2011; File et al., 2022; Shilpa et al., 2017) (Please see reference no. 45,22,16 ). These studies show that even one organic input can markedly enhance nutrient efficiency and soil biological activity when used with balanced mineral fertilizers. Moreover, the national chili production guideline described in Krishi Projukti Hatboi (BARI Publication) [https://drive.google.com/file/d/1ErE_Fmhs2v_b9xgLzKEuE_5dPoAVlerF/view?usp=sharing] also recommends six inorganic fertilizers such as urea, triple superphosphate, muriate of potash, gypsum, zinc sulfate and boric acid-in varying doses for standard chili cultivation in Bangladesh. Furthermore, our mixed approaches aimed to observe whether addition of organic fertilizer reduce the uses of inorganic fertilizer compared to recommended doses of inorganic fertilizer which otherwise maintain the soil health for long term. Importantly, our fertilizer combination followed this recommended baseline, with L. leucocephala leaf litter added as the organic component to improve nutrient synchronization and soil quality. Regarding our title, we humble inform you that, our current research not only evaluate the role of L. leucocephala leaf litter but evaluate/compare the effect of sole L. leucocephala leaf litter with combined mixed approaches (L. leucocephala + RDF) for chili production thus all potential co-authors agree to keep both “mono and mixed fertilization” in the title section. In response to the reviewer’s suggestion, we added the role of each inorganic fertilizer. (Please see L 152-158).*

**Reviewer Comment**

In the Results section: 3.1 Growth Dynamics 3.2 Reproductive traits and so on.

**Author Response**

*We appreciate this structural suggestion. The headings of each paragraph in the result section have been updated based on the figures. Following reviewer suggestion we revised our figures, for example, figure 3 showed the effect of treatments on vegetative and reproductive traits of chili depends on different days after transplantation (DAT). Hence, this figure 3 showed the changes of both vegetative and reproductive traits in across different growth stages. Therefore, we updated the title in line with figure 3 like “3.1- Mixed fertilization enhances vegetative and reproductive traits of chili across different growth stages” (Please see L 231-232). As we addressed the effect of treatments on reproductive traits of chili in figure 3, thus separately mentioned “Reproductive Trait” as title has been diminished. Similarly, we also revised some other figures such as 5 and 6 (previously figure 8 and figure 9 respectively) based on reviewer suggestion and also revised the titles based on actual suitability. Please see the title number 3.3, 3.4 (previously section 3.4 and section 3.5 respectively) and also the caption of figure 5, 6.*

**Reviewer Comment**

Discussion portion should be reduced and more focus should be given on the comparison of chemical composition between organic and inorganic fertilizers to explain the improved plant growth and other studied parameters. Physiological and biochemical changes in the plant due to addition of organic manure should also be included.

**Author Response**

***We appreciate the reviewer’s comment. The discussion section has been revised and shortened (please see the excluded line in tract change file L 430-431, 436-438, 463-465, 482-483, 490-491) according to the reviewer comments focusing the direct interpretation of our results. Firstly, our chemically analyzed quality traits such as vitamin C, capsaicin content and SPAD reading represented how organic, inorganic and mixed fertilizer application influence the above mentioned parameters, where we found each treatment showed significant change effect on the quality traits of chili (Please see L 277-285). Secondly, our result also revealed that mixed fertilizer treatment represent improved quality traits compared to other treatments and we sufficiently interpreted the reasons behind our results in discussion section (Please see L 371-386). Finally, we justify and interpret the results of physiological responses in the discussion section. (Please see L 314-344).***
