# Supplementary material for: Beyond mono fertilization: Mixed fertilization enhances productivity and quality of chili (Capsicum frutescens)
Source: PLoS One. 2026 Mar 31;21(3):e0331538. doi: 10.1371/journal.pone.0331538 (PMC13037990; doi:10.1371/journal.pone.0331538)
Supplement: Attachment — Submitted filename: Response to Reviewers Comments.docx [file pone.0331538.s005.docx]

**Response to Editor**

**◘ Editor Comment**

Address the remaining points from Reviewer #1

**Author Response**

*We appreciate your guidance. All remaining comments from Reviewer #1 have been carefully addressed in the revised manuscript accordingly. Please refer to the response to reviewer comments#1 given below and also see our revised manuscript.*

**◘ Editor Comment**

Further edit the abstract by reducing the number of individual values and p-values. Emphasize the main effect sizes and trends.

**Author Response**

*Thank you very much for your valuable comment. The Abstract has been revised to improve clarity and readability by reducing the number of individual numerical values and p-values. Instead of listing multiple statistical details, the revised abstract now emphasizes the overall effect sizes, major trends and key findings of the study. Our revised manuscript is now more improves in terms of narrative flow and highlights the primary outcomes of the research more clearly (please see L 28-29, 31-36). Revised abstract is now shown as:*

*“While the effects of different fertilization strategies on chili (Capsicum frutescens) cultivation have been previously examined, the lack of comparative assessment of mixed versus mono-fertilization approaches limits our understanding. To address this gap, we conducted an experiment using a completely randomized design (CRD) with five fertilization treatments along with four replications of each treatment (e.g., Leucaena leucocephala leaf litter and recommended dose of synthetic fertilizers (RDF)) to observed the effect of these treatments on productivity traits, NPP (net primary productivity), quality, and overall yield of chili. We found that mixed fertilization (T_4_) results higher yield (~ 60% and ~ 90%) compared to control (T_0_) and sole organic fertilizer (T_1_+T_2_) respectively. The T_4_ treatment showed consistently higher plant height, leaf and fruit number at 60 DAT, as well as higher flowering at 45 DAT compared to other treatments. We also found that NPP (above- and belowground combined) was higher in T_3_ and T₄ treatments compared to control and organic fertilization. Similarly, quality traits such as vitamin C, capsaicin content, and SPAD reading were higher under mixed fertilization. Linear fit regression model indicated that both ANPP and yield were positively associated with vegetative and reproductive traits, particularly leaf number and flowering, highlighting that structural growth directly contributed to productivity gains. Overall, our results suggest that mixed fertilization enhances both productivity and quality traits of chili. Therefore, integrated organic and inorganic fertilizer management are recommended to improve yield and quality of chili.”*

**◘ Editor Comment**

Explicitly highlight in the Discussion and Conclusions that the fertilizer-treatment set is not a complete factorial of organic × inorganic levels and that this limit what can be inferred (mention what effects cannot be estimated). This is mentioned, but only once.

**Author Response**

*Thank you for your comments. The Discussion and Conclusions sections have been revised and explicitly clarify that the fertilizer treatment structure does not represent a full factorial design of organic × inorganic fertilizer levels. As a result, the study cannot statistically estimate the independent main effects of organic and inorganic fertilizer treatment. The observed differences therefore represent comparisons among specific treatment combinations used in this study rather than a complete factorial analysis. As suggested, this limitation is now clearly stated in both the Discussion and Conclusions sections, and future research using a complete factorial design is also recommended (please see L 432-441and L 460-463).*

**◘ Editor Comment**

Replace all speculative phrases (“we assume… may enhance”) with either a clear indication that these are hypotheses for future work or citations that support the proposed mechanisms.

**Author Response**

We apologize for using such speculative phrases. In the current form, we carefully reviewed the discussion section and replace all speculative phrases as suggested. Example of some revised sentences are:

***Original text (speculative):*** *“Although we did not measure the mechanism behind the findings, however, considering other experiment, we assumed that the presence of organic matter such as L. leucocephala leaf litter enhances rhizospheric activity and microbial interactions that release essential growth hormones like cytokinins and auxins.”*

***Revised text:*** *‘Previous studies have also reported that the presence of organic matter such as L. leucocephala leaf litter may enhance rhizospheric microbial activity and nutrient-mediated hormonal processes that influence flower retention and fruit development [13,14]. However, direct measurements of microbial dynamics and hormone regulation would be required to confirm these pathways’ (L 350-354). We also excluded those speculative words such as “could be due to, supposed to, this is because, although……. may enhances” in the revised text (please see L 369-372, 378-382, 384-388, 398-401).*

**Response to Reviewer**

**Response to Reviewer comments # 1**

**◘ Reviewer Comment**

The document improved from the previous version, but the authors still must make corrections in the text. For example, the document needs an extensive English revision.

**Author Response**

*We thank for your meaningful suggestion. The entire manuscript has now undergone a comprehensive English language revision using advanced grammar and style correction tools. In addition, all the co-authors of this manuscript carefully reviewed sentence structure, tense consistency, subject–verb agreement, clarity of expression, and scientific phrasing throughout the manuscript. We believe, the current revised version reflects substantial improvement in readability, fluency, and grammatical accuracy. Please refer to our manuscript.*

**◘ Reviewer Comment**

There are no results or discussion for the whole section ‘2.2. Pot preparation and experimental design.

**Author Response**

*Thank you for this observation. Section 2.2. Pot preparation and experimental design belong to the ‘Materials and Methods’ section and therefore we clearly described the details experimental procedures in the Materials and Methods rather than presenting results section. The results section substantially described our research findings.*

*However, to improve clarity and avoid possible confusion, we carefully revised this section to provide a clearer description of the experimental layout, pot preparation procedure, treatment structure, and replication used in the study. For example, see L 117-121, L 146-148. These revisions ensure that the experimental design is more clearly explained to readers.*

**◘ Reviewer Comment**

The introduction contains many ideas within only two paragraphs. The reviewer suggests dividing the content into smaller paragraphs structured as follows:

- Importance of chili
- Importance of mineral nutrition with its shortcomings
- Importance of organic fertilizers with their shortcomings
- Advantages of combining both fertilizer types
- Objectives of the study

**Author Response**

*Thank you for your helpful and structural suggestion. As suggested, the Introduction has now been reorganized and clearly defined paragraphs following exactly the recommended structure:*

1. *Importance of chili production (please see L 43-51)*
2. *Importance and limitations of mineral nutrition (L 52-57)*
3. *Role and limitations of organic fertilizers (L 58-64)*
4. *Rationale for integrated nutrient management (L 65-82)*
5. *Clear statement of study objectives (L 83-91)*

**◘ Reviewer Comment**

Please explain the meaning of an abbreviation the first time it is written in the text. Therefore, please explain the meaning of TSP and MOP in line 124, not 149–150.

**Author Response**

*Thank you for your comments. The abbreviations TSP and MOP are now fully defined at their first appearance in Section 2.2:*

- *TSP is defined as Triple Super Phosphate. (L 127)*
- *MOP is defined as Muriate of Potash. (L 127)*

**◘ Reviewer Comment**

There is no result regarding the decomposition kinetics of the leucaena leaves. Please add this information to the document

**Author Response**

*We appreciate this observation. The litterbag technique in this study was primarily used to ensure gradual nutrient release from* ***Leucaena leucocephala*** *leaf litter during the experimental. Now, we have revised and added a brief explanation of the decomposition dynamics in “2.2.2. Application of litterbag technique’’ section. We followed the negative exponential decay model to assess decomposition dynamics of L. leucocephala leaf litter (Olson, 1963). Please see L 173-177, 183-186.*

**◘ Reviewer Comment**

Please pay attention to scientific names such as “*L. leucocephala*.” The species name must always be written in lowercase.

**Author Response**

*We apologies for this issue. All scientific names have been carefully checked throughout the manuscript as advised. The genus and species name* ***Leucaena leucocephala*** *is now consistently written following standard scientific nomenclature, with the genus capitalized and the species epithet in lowercase.*
